# Gene and Cellular Therapies for Leukodystrophies

**DOI:** 10.3390/pharmaceutics15112522

**Published:** 2023-10-24

**Authors:** Fatima Aerts-Kaya, Niek P. van Til

**Affiliations:** 1 Department of Stem Cell Sciences, Graduate School of Health Sciences, Center for Stem Cell Research and Development, Hacettepe University, 06100 Ankara, Turkey; fatima.aerts@hacettepe.edu.tr; 2 Advanced Technologies Application and Research Center, Hacettepe University, 06800 Ankara, Turkey; 3 Amsterdam Leukodystrophy Center, Emma Children’s Hospital, Amsterdam University Medical Centers, Amsterdam Neuroscience, 1081 HV Amsterdam, The Netherlands; 4 Department of Integrative Neurophysiology, Center for Neurogenomics and Cognitive Research, Vrije Universiteit Amsterdam, 1081 HV Amsterdam, The Netherlands

**Keywords:** leukodystrophies, gene therapy, stem cell transplantation, lentiviral vectors, adeno-associated viral vectors

## Abstract

Leukodystrophies are a heterogenous group of inherited, degenerative encephalopathies, that if left untreated, are often lethal at an early age. Although some of the leukodystrophies can be treated with allogeneic hematopoietic stem cell transplantation, not all patients have suitable donors, and new treatment strategies, such as gene therapy, are rapidly being developed. Recent developments in the field of gene therapy for severe combined immune deficiencies, Leber’s amaurosis, epidermolysis bullosa, Duchenne’s muscular dystrophy and spinal muscular atrophy, have paved the way for the treatment of leukodystrophies, revealing some of the pitfalls, but overall showing promising results. Gene therapy offers the possibility for overexpression of secretable enzymes that can be released and through uptake, allow cross-correction of affected cells. Here, we discuss some of the leukodystrophies that have demonstrated strong potential for gene therapy interventions, such as X-linked adrenoleukodystrophy (X-ALD), and metachromatic leukodystrophy (MLD), which have reached clinical application. We further discuss the advantages and disadvantages of ex vivo lentiviral hematopoietic stem cell gene therapy, an approach for targeting microglia-like cells or rendering cross-correction. In addition, we summarize ongoing developments in the field of in vivo administration of recombinant adeno-associated viral (rAAV) vectors, which can be used for direct targeting of affected cells, and other recently developed molecular technologies that may be applicable to treating leukodystrophies in the future.

## 1. Leukodystrophies

Leukodystrophies are a heterogenous group of inherited, degenerative encephalopathies, that, due to their primary involvement of the central nervous system (CNS) white matter, are often diagnosed by their characteristic changes in white matter imaging. Although patients are often young, onset of the diseases may occur at any age [1], depending on the genetic defect and residual protein activity. Clinically, leukodystrophies cause motor dysfunction and varying degrees of hypotonia/spasticity that may increase progressively in time. This often results in a low quality of life and high fatality rates, since a pertinent curative treatment has not yet been developed [2]. In contrast to the general belief that most leukodystrophies are caused by mutations in myelin- or oligodendrocyte-specific genes, a substantial fraction of the disease is related to pathogenic variants in other white matter components, such as astrocytes, microglia, neuronal axons, and brain vasculature [1], or may be caused secondary to accumulation of unprocessed metabolic derivatives. In addition, it has recently been shown that gene defects that affect DNA/RNA transcription/translation can result in white matter pathology and leukodystrophies as well [3].

Currently, more than 30 distinct types of leukodystrophies have been classified [4], and although the incidence of each individual leukodystrophy is low, together these leukodystrophies may affect as many as 1:7500 persons [5].

## 2. Classification and Treatment of Leukodystrophies

Classical (historical) classification of leukodystrophies is usually based on the presence of specific radiographic MRI findings, classifying the diseases in demyelinating, hypomyelinating, spongiform and cystic disorders [4,6]. However, recent classification based on cellular pathology has gained more interest due to the rapid increase and advances in molecular diagnostics. Using this system, it has been proposed to classify the leukodystrophies into myelin disorders (including disorders affecting oligodendrocyte and myelin), astrocytopathies, leuko-axonopathies, microgliopathies and leuko-vasculopathies [1]. An overview of pediatric leukodystrophies is shown in Table 1. 

Generally, treatment options for most leukodystrophies are limited to supportive treatments, with the exception of cerebrotendinous xanthomatosis (CTX), which due to the nature of the disease (lipid metabolism), can be managed using chenodeoxycholic acid (CDCA) replacement therapy in combination with HMG-CoA reductase inhibitors. For other leukodystrophies, medication is often used to relieve symptoms related to dystonia or seizures, whereas nutritional support may be needed to manage dietary restrictions and suppletion in these patients. Allogeneic hematopoietic stem cell/hematopoietic stem and progenitor cell (HSC/HSPC) transplantation for the treatment of leukodystrophies has been associated with high mortality and high morbidity, due to transplantation/chemotherapy-related issues, such as graft-versus-host disease, protracted infections and organ damage [7]. In addition, not all leukodystrophies are eligible for HSC transplantation.

The largest group of HSC transplantations for leukodystrophies has been performed for the treatment of globoid cell leukodystrophy (GCL, Krabbe), X-linked adrenoleukodystrophy (X-ALD) or metachromatic leukodystrophy (MLD). These studies all used unrelated umbilical cord blood (UCB) as a source of HSCs and generally show that early UCB-HSC transplantation is beneficial in terms of survival and maintenance of cognitive and motor function, especially when performed before the onset of clinical symptoms [8,9,10]. However, when UCB-HSC transplantation is performed in already symptomatic patients, these patients will generally continue to deteriorate and eventually die prematurely. The use of unrelated UCB as a source of HSCs has two advantages over allogeneic-related or haplo-identical transplants. In the first place, cord blood banks provide off-the-shelf use of UCB, which can be used immediately after diagnosis without inducing further delay. Secondly, since related allogeneic donors may be possible disease carriers and clinical outcome has been shown to be correlated to protein expression levels, these donors are not preferred [11]. In allogeneic donor transplantation, endogenous normal wildtype gene expression determines efficacy, and cross-correction may not occur if therapeutic levels are insufficient [12]. In contrast, gene therapy could provide supraphysiological levels, substantially enhancing therapeutic efficacy.

Gene therapy for presently untreatable inborn errors of metabolism, genetic neurodegenerative diseases and immune deficiencies has gained considerable interest in the last 20 years. Currently, over 3500 gene therapy trials worldwide have been registered according to the Journal of Gene Medicine (Wiley), of which 1.7% (64 clinical trials) are focusing on neurological diseases [13]. However, these also include clinical trials for the treatment of neurodegenerative diseases, such as Parkinson’s, Alzheimer’s and Huntington’s, amyotrophic lateral sclerosis, multiple sclerosis, myasthenia gravis, and diabetic neuropathy, and only a handful of clinical trials have been undertaken or are currently ongoing/recruiting to address the treatment of inherited neurodegenerative diseases, such as mucopolysaccharidosis III (Sanfilippo syndrome) and leukodystrophies (ALD, MLD and GLD) (summarized in Table 2).

However, prospects for treatment and diagnosis of leukodystrophies are rapidly increasing due to the discovery of genes involved, a better understanding of the pathophysiology of the diseases, earlier diagnosis and improved diagnostic tools, as well as the development of treatment modalities using gene replacement, gene addition or genetic correction. This article will focus on some of the leukodystrophies that show the greatest potential for slowing down ongoing neurodegeneration, maintenance of cell function and/or improvement using interventional gene therapy.

**Table 1:** Classification and characteristics of the different types of pediatric leukodystrophies.

Disease	Affected Gene/ Protein	Inheritance	Prevalence	Affected System	Historical (Radiographical) Classification [14]	Functional Classification
***Inborn errors of metabolism***						
X-linked adrenoleuko- dystrophy (X-ALD) [15]	*ABCD1/*ATP binding cassette, subunit D	X-linked (female carriers may be affected)	1/14,000–17,000 in males	Peroxisome, lipid metabolism	demyelination	myelin disorder (demyelination)
Globoid cell leukodystrophy (Krabbe)	*GALC/* Galactosyl- ceramidase	Autosomal recessive	1–5/100,000	Lysosome, lipid metabolism	demyelination, myelin vacuolization	myelin disorder (demyelination)
Metachromatic Leukodystrophy (MLD)	*ARSA/* Arylsulfatase A	Autosomal recessive	1/40,000–160,000	Lysosome, lipid metabolism	demyelination	myelin disorder (demyelination)
Fabry disease (FD) [16]	*GLA/* α-galactosidase A	X-linked (female carriers may be affected)	1/20,000–40,000	Lysosome, Lipid metabolism	hypomyelination	Secondary (glycosphingo- lipid deposition)
Cerebrotendinous Xanthomatosis (CTX) [17]	*CYP27A1/* mitochondrial enzyme sterol 27-hydroxylase	Autosomal recessive	1/50,000–1,000,000	Lipid (Cholesterol) metabolism	cerebellar and cerebral atrophy	Secondary (cholesterol- derivative accumulation)
Sjögren-Larsson Syndrome (SLS) [18]	*FALDH (ALDH3A2)/* Fatty aldehyde dehydrogenase	Autosomal recessive	1/250,000	Lipid metabolism	cerebral atrophy	Secondary (accumulation of fatty alcohols and fatty aldehydes)
Pompe disease [19]	*GAA/* acid α-glucosidase	Autosomal recessive	1/40,000	Lysosome, Glycogen metabolism	demyelination	Secondary (glycogen accumulation)
Canavan disease	*ASPA*/ aspartoacylase	Autosomal recessive	1/100,000	Absence of myelin lipid synthesis	spongiform (myelin vacuolization)	myelin disorder (vacuolization)
Peroxisomal biogenesis disorders (Zellweger syndrome, neonatal leukodys-trophy and infantile Refsum disease) [20]	*PEX1/*Peroxisomal biogenesis factor 1	Autosomal recessive	1/50,000	Peroxisome assembly	demyelination	myelin disorder
***Disorders of RNA/DNA Transcription/Translation***						
Congenital peripheral hypomyelinating neuropathy, central dys- myelination and Waardenburg–Hirschsprung (PCHW) [21]	*SOX10/*SOX10	Autosomal dominant	<1/ 1,000,000	Myelin development	hypo- myelination	myelin disorder [22]
Aicardi-Goutieres syndrome [23]	*ADAR, TREX1, RNASEH2A, RNASEH2B, RNASEH2C, SAMHD1, IFIH1*	Autosomal recessive or dominant	1–5/10,000	Nuclease genes	intracerebral calcifications, cerebral atrophy, temporal cysts	astrocytopathy
Childhood ataxia with CNS hypomyelination/ Vanishing White Matter disease (CACH/VWM) [24]	*EIF2B1-5*/translation initiation factor eIF2B subunits	Autosomal recessive	1–4/ 1,000,000 births	isolated oligodendrocyte and astrocyte cell death	hypo- myelinating	astrocytopathy
***Cytoskeletal***						
Alexander disease [25,26,27]	*GFAP/*glial fibrillary acidic protein	Autosomal dominant	1/ 1,000,000 births	accumulation of GFAP in Rosenthal fibers	hypo- myelination, spongiform	astrocytopathy
Hypomyelinating leukodystrophy with atrophy of the basal ganglia and cerebellum (H-ABC) [28]	*TUBB4A/*tubulin β-4A	Autosomal dominant	unknown	alteration of microtubule dynamics or stability	hypomyelination, small or absent putamen, cerebral and cerebellar atrophy	leuko-axonopathy
***Myelin disorders***						
Pelizaeus-Merzbacher disease (PMD, HLD1)	*PLP1/*proteolipid protein	X-linked	1/100,000	Myelin protein disorder	diffuse hypo- myelination, spongiform	myelin disorder (hypomyelination)
Pelizaeus-Merzbacher-like disease (PMLD) [22]	*GJC2/*gap-junction protein, gamma-2 and others (*HSPD1, FAM126A, POLR3A, POLR3B, RARS, PYCR2, POLR1C,* *VPS11, SLC16A2)*	Autosomal recessive	unknown	Gap junctions	diffuse hypo- myelination	Myelin disorder (hypomyelination)
Hypomyelinating leukodystrophy (HLD3) [29]	*AIMP1/*ARS-interacting multifunctional protein 1	Autosomal recessive	unknown	Hyperphosphorylation of neurofilament proteins	cerebral atrophy, hypomyelination	leuko-axono- pathy
Fucosidosis [30]	*FUCA1*/alpha fucosidase	Autosomal recessive	<1/200,000 births	Lysosome	hypomyelination, cerebral and cerebellar atrophy	leuko-axono- pathy (oligo- dendrocyte)
Hypomyelination with congenital cataract (HCC) [31,32]	*FAM126A* (*DRCTNNB1A*)/ Hyccin	Autosomal recessive	Very rare, unknown	Myelin production	hypomyelination, white matter atrophy	leuko-axono- pathy (oligo- dendrocyte)

**Table 2:** Overview of gene therapy clinical trials for primary leukodystrophy.

Clinical Trial	Title/Year	Country	Vector/ Transgene	NTC No
Ex vivo gene therapy				
Phase I/II	Gene therapy for metachromatic leukodystrophy (MLD) (2010–2025)	Italy	SIN-LV-ARSA (Libmeldy)	NCT01560182
Phase II/III completed	A Study of the efficacy and safety of Hematopoietic Stem Cells transduced with Lenti-D lentiviral vector for the treatment of cerebral adrenoleukodystrophy (CALD) (2013–2021)	USA	Lenti-D-ABCD1 (Skysona)	NCT01896102
Phase I/II recruiting	Autologous Hematopoietic Stem Cell Gene Therapy for Metachromatic Leukodystrophy and Adrenoleukodystrophy (2015–2025)	China	LV-ARSA/ LV-ABCD1	NCT02559830
Phase II	A Safety and Efficacy Study of Cryopreserved OTL-200 for Treatment of MLD (2018–2028)	Italy	LV-ARSA	NCT03392987
Phase I/II	Lentiviral gene therapy for MLD (2018–2020)	China	LV-TYF-ARSA	NCT03725670
Phase I/II	Lentiviral Gene Therapy for X-ALD (2018–2020)	China	LV-TYF-ABCD1	NCT03727555
Phase III	A Clinical Study to Assess the Efficacy and Safety of Gene Therapy for the Treatment of Cerebral Adrenoleukodystrophy (CALD) (2019–2023)	USA	Lenti-D-ABCD1 (Skysona)	NCT03852498
Phase III recruiting	OTL-200 in Patients With Late Juvenile Metachromatic Leukodystrophy (MLD) (2022–2031)	Italy	SIN-LV-ARSA (Libmeldy)	NCT04283227
In vivo gene therapy				
Phase I/II	Intracerebral Gene Therapy for Children With Early-Onset Forms of MLD (TG-MLD) (2014–2029)	France	AAVrh10-ARSA	NCT01801709
Phase I/II	rAAV-Olig001-ASPA Gene Therapy for Treatment of Children With Typical Canavan Disease (CAN-GT) (2021–2024)	USA	rAAV-Olig001-ASPA	NCT04833907
Phase I/II recruiting	Gene Transfer Clinical Trial for Krabbe Disease (RESKUE) (2021–2024)	USA	AAVrh10-GALC	NCT04693598
Phase I/II recruiting	A Study of AAV9 Gene Therapy in Participants With Canavan Disease (CANaspire) (2021–2028)	USA	rAAV9-ASPA	NCT04998396
Phase I/II	Study of Safety, Tolerability and Efficacy of PBKR03 in Pediatric Subjects With Early Infantile Krabbe Disease (GALax-C) (2022–2030)	USA	AAVHu68-GALC	NCT04771416
Phase I/II recruiting	Gene Transfer Clinical Trial for Infantile and Late Infantile Krabbe Disease Treated in the Past With HSCT (REKLAIM) (2023–2025)	USA	AAVrh10-GALC	NCT05739643

### 2.1. X-Linked Adrenoleukodystrophy (ALD)

The first mention of adrenoleukodystrophy in the medical literature dates back to 1897 [33], whereas evidence for X-linked inheritance was first described almost a century later [34]. X-linked adrenoleukodystrophy (X-ALD) was shown to be caused by mutations in the *ABCD1* gene. This gene encodes an ABD transporter protein (ATP-binding cassette, subunit D, ALD), which is localized in the peroxisomal membrane and is necessary for oxidation and metabolism of very-long-chain fatty acids (VLCFA) [35]. Deficiency of the ALD protein leads to storage of these VLCFA in plasma, adrenal glands, testes and brain tissue and causes progressive demyelination of the CNS [4,36]. X-ALD can be classified into different types of ALD (e.g., childhood cerebral X-ALD, adolescent cerebral X-ALD, adult cerebral ALD, adrenomyeloneuropathy or Addison-disease only) based on the initiation of symptoms and involvement of different tissues [37]. Childhood cerebral X-ALD is the most severe form of ALD and typically affects boys at the age of 5–12 years, resulting in rapidly progressive neurodegeneration and death within 5 years [38,39]. The first HSC transplantation for treatment of X-ALD was performed approximately 3 years before the discovery of the *ABCD1* gene and showed the feasibility of reversing early neurological symptoms [40]. Nevertheless, the use of bone marrow transplantation (BMT) for childhood cerebral X-ALD only became common after long-term results on prognosis and survival were published [41]. In addition to BMT, the use of Lorenzo’s oil (4:1 glyceryl trioleate-glyceryl trierucate) was recommended in asymptomatic boys with X-ALD and normal brain MRI to reduce plasma levels of VLCFA and prevent disease progression [42,43]. 

The results from the first lentiviral HSPC gene therapy trial for treatment of X-ALD in two patients were published in 2009, and showed that 1 year after gene therapy, progressive cerebral demyelination had been halted, similar to treatment with allogeneic HSC transplantation [44]. In another trial for cerebral X-ALD, the effects of Lenti-D gene therapy were assessed in 17 boys. Early results showed measurable ALD protein levels, absence of integrations near known oncogenes, and a success rate of 88% in terms of survival and absence of functional disability, suggesting that the lentiviral gene therapy approach is safe and efficacious [7]. 

### 2.2. Metachromatic Leukodystrophy (MLD)

Although sporadic reports of progressive cerebral sclerosis in infants were presented in the medical literature before, the first clinical and histopathological description of two children fitting the diagnosis of metachromatic leukodystrophy (MLD) or Greenfield’s disease was made in 1933. Here, Greenfield specifically described the wide spread demyelination of white matter, absence/degeneration of oligodendrocytes and accumulation of abnormal granules [45]. The role and involvement of sulfatases in the development of MLD was revealed in the 1960s [46,47] and was followed by the identification of arylsulfatase A (ARSA, also known as cerebroside-sulfatase) as the responsible gene [48]. Deficiency of ARSA, which has now been shown to cause accumulation of sulfatides (both galactosyl and lactosyl sulfatide) in white matter, results in progressive loss of motor and cognitive skills. However, the rate of deterioration is dependent on residual ARSA enzyme activity and may change depending on the type of mutation [49,50]. In a smaller fraction of patients, mutations in the PSAP gene, which codes for the lysosomal sphingolipid activator protein B (SapB or saposin B), have been found to be responsible for the development of MLD [51]. Based on the first appearance of symptoms and diagnosis, three different types of MLD have been recognized, i.e., late-infantile (<2 years), juvenile (3–15 years) and adult (>16 years) [52].

The first bone marrow transplantations for the treatment of MLD were performed early in the 1980s [53], aiming at metabolic cross-correction with donor-derived ARSA-expressing hematopoietic cells. Since then, many patients have been treated with HSC transplants from mainly bone marrow and umbilical cord blood. Although the overall results show a delayed loss of motor and cognitive functions post HSC transplantation, the highest efficacy of HSC transplant can be obtained in juvenile MLD in comparison with infantile MLD, especially when transplants are performed at an early, pre-symptomatic stage [54,55,56,57]. Autopsy brain tissue material obtained after HSC transplantation for MLD revealed the presence of ARSA-expressing donor-derived tissue macrophages dispersed throughout white matter and evidence of remyelination. However, no evidence was found for the cross-correction of astrocytes and oligodendrocytes [12,58], indicating that allogeneic HSC transplantation slows disease progression, but local ARSA expression by donor cells is not sufficient for metabolic correction of MLD. Higher expression may be required for efficient cross-correction to enhance secretion into the extracellular space. In addition to HSC transplantation, the use of allogeneic mesenchymal stromal/stem cells (MSC) has also been proposed, since MSCs have been shown to be able to migrate and engraft in the brain, differentiate into astrocytes, and express high levels of ARSA [59,60,61]. When MSCs were infused in patients who had persistent progressive neurologic and skeletal defects despite achieving 100% donor chimerism after HLA-identical allogeneic bone marrow transplantation, patients displayed increased bone mineral density and improved nerve conduction velocity, but no clinical improvement in overall health, or mental and physical development [62]. Similarly, when patients were treated with MSC infusions shortly after allogeneic HSC transplantation, they showed improved hematopoietic reconstitution and increased *ARSA* expression, but no overall improvement in clinical outcome and gross motor function [63].

*ARSA* overexpression using gene therapy could overcome these issues. The physiological phosphoglycerate kinase (PGK) promoter has shown a lower genotoxic risk compared to the use of stronger viral promoters, such as the spleen focus-forming virus (SFFV) promoter or the MND promoter [64,65]. A clinical phase 1/2 gene therapy trial for MLD patients using the PGK promoter showed early beneficial effects with high levels of ARSA activity in the peripheral blood after transplantation in patients with autologous CD34+ HSCs encoding *ARSA* gene after ex vivo lentiviral transduction [66]. Long term results from this study showed improved survival, locomotor function and persistent high-level *ARSA* expression in peripheral blood [66,67,68]. Furthermore, most patients displayed a normal cognitive development and a prevention/delay of central and peripheral demyelination and brain atrophy throughout the follow up. Treatment benefits were particularly apparent in patients treated before the onset of symptoms. After busulfan conditioning, all treated patients showed sustained multilineage engraftment of genetically modified HSPCs, in the absence of abnormal cellular proliferation [67]. In 2020, this gene therapy product was approved by the EMA as atidarsagene autotemcel (Libmeldy, OTL-200, Arsa-cel) for the treatment of children with late infantile or early juvenile forms of MLD, who are presymptomatic or have not yet developed mental deterioration [69]. Additional approval by the FDA is expected later this year, since the biological license application by Orchard therapeutics has been completed.

### 2.3. Globoid Cell Leukodystrophy (GCL, Krabbe)

In 1916, Krabbe described a genetically inherited infantile form of diffuse brain sclerosis that is now known as Krabbe disease or globoid cell leukodystrophy, due to the presence multinucleated large cells in both the CNS and the peripheral nervous system [70]. GCL is a rare, autosomal recessive inherited lysosomal storage disorder caused by a deficiency of the acid hydrolase, galactosylceramidase (GALC) [71,72,73]. GALC degrades galactosylceramides and sphingolipids, and in the absence of a functional enzyme toxic accumulation of galactosylsphingosine in oligodendrocytes and Schwann cells, causes progressive demyelination of the CNS and the peripheral nervous system (PNS) [74,75,76]. Krabbe disease can be divided into infantile, juvenile and adult-onset forms. The infantile form of Krabbe disease is the most common form of GCL and is characterized by a rapidly progressive clinical decline and death between the ages of 2–4 years if left untreated. Allogeneic HSC transplantation has been shown to be effective and restores leukocyte galactocerebrosidase levels [77]. However, although CNS deterioration can be partially reversed or delayed, it cannot be completely stopped, particularly if PNS involvement is not effectively treated [78]. This has been linked to an insufficient uptake of donor cell-secreted GALC by the mannose 6-phosphate-receptor [79]. Therefore, different types of treatments, such as enzyme replacement therapy (ERT), substrate reduction therapy (SRT), the use of pharmacological chaperone therapy (PCT) for the treatment of missense mutations [80], and gene therapy, are being developed. Using a lentiviral vector encoding a codon-optimized human *GALC* gene expression by HSCs could be efficiently obtained. However, overexpression of the *GALC* gene in HSCs has been linked to cellular toxicity, which has important implications for vector design and fine-tuning of protein expression [81]. A combination of physiological promoters or lineage- (differentiation) specific promoters, such as myeloid-specific enhancer/promoter sequences for CD11b cells, in combination with microRNA (miR-126) target sequences to reduce expression in HSCs has shown successful engraftment, reconstitution and correction in the Krabbe twitcher mouse [82,83]. In addition, the use of GALC fusion proteins to bypass the mannose-6-phosphate-dependent uptake may result in increased cellular uptake and cross-correction of affected cells [79]. Due to these backlashes, clinical trials for the treatment of GCL have been more focused on the testing and development of rAAV vectors (see below) and have not yet been approved for clinical use. 

**Figure 1:**
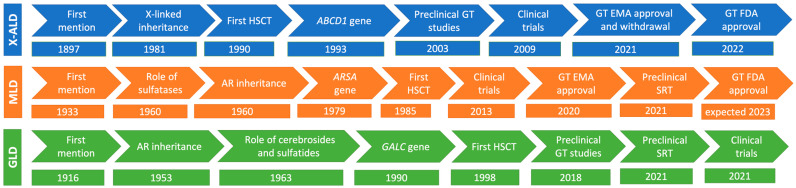
Timelines of ALD, MLD and GLD: from recognition to the development of gene therapy. Development of gene therapy for the major primary leukodystrophies has taken well over a century to reach clinics. However, it is expected that the development and approval of gene therapy for similar diseases may be much faster since most of the footwork has been carried out and many of the pitfalls have been revealed and tackled in subsequent studies. AR: autosomal recessive; GT: gene therapy.

A timeline depicting the developments from first description of the diseases in the medical literature to current (gene therapy) treatments for X-ALD, MLD and GCL is shown in Figure 1.

## 3. Gene Therapeutic Approaches to Treat Early-Onset Leukodystrophies

### 3.1. Issues Regarding the Development of HSPC Gene Therapy for the Treatment of Leukodystrophies

Clinical issues have been raised with respect to long-term safety, since the use of gammaretroviral vector-modified HSC transplants have been associated with insertional mutagenesis due to viral integrations near proto-oncogenes, resulting in the development of leukemias [84]. However, the third generation, self-inactivating lentiviral vectors that have been used in multiple clinical trials for X-ALD [36], MLD [66,68], β-thalassemia [85] and Wiskott-Aldrich syndrome [86] have been shown to be considerably safer. Nevertheless, a case of acute myeloid leukemia (AML) was reported after treatment with a LentiGlobin vector for the treatment of sickle cell anemia [87], as well as three cases of myelodysplastic syndrome (MDS) after treatment with Eli-cel. The latter is likely linked to the lentiviral vector, which integrated near proto-oncogenes *MECOM* and *PRDM16* and contained specific features such as a strong MND promoter [88,89,90]. However, preclinical assessment did not identify insertional mutagenesis as a quantifiable hazard, and the clinical study highlighted the importance of long-term follow up, assessing vector design elements in disease-specific contexts to decipher factors that contribute to these MDS cases [90]. Accordingly, both the EMA (July, 2021) and the FDA (September, 2022) approved the use of elivaldogene autotemcel (Lenti-D, Skysona, Eli-cel) to slow the progression of neurologic dysfunction in adolescent boys with early, active cerebral ALD, believing the benefits of the treatment outweigh the currently known risks to patients who have no available HLA-matched donor [89,91,92]. Risks for the development of MDS or leukemia not only depend on the vector, but also on the promoter and the transgene used; therefore, risk benefits need to be assessed on a disease-to-disease basis.

Secondly, issues have been raised regarding which type of conditioning should be offered to patients before the transfer of genetically modified autologous CD34+ HSPCs. Although conditioning regimens used in HSPC gene therapy are generally more benign than in allogeneic HSPC transplantation, often using non-myeloablative regimens, the alkylating agents used are still toxic. More gentle approaches that create enough space in the bone marrow for genetically modified HSPCs to engraft are still needed [93]. In recent times, a number of innovative proof-of-concept approaches for antibody-mediated pre-conditioning have emerged. These methods have the ability to specifically target HSCs/HSPCs and immune cells, while keeping overall toxicity to a minimum. Utilizing antibody–drug conjugates in conjunction with either reduced-intensity conditioning or high-dose antibody–drug conjugate treatment as a stand-alone therapy has demonstrated potential for allo-HSCT in preclinical studies [94]. 

Lastly, as described above, lentiviral vectors have been developed and clinically tested for X-ALD and MLD, resulting in the clinical approval of two lentiviral gene therapy products. The development of lentiviral vectors for the treatment of GCL is still ongoing due to issues regarding cytotoxicity, insufficient cellular uptake and cross-correction of affected cells. Another issue that need to be addressed is the reimbursement of costs by insurance companies or governments: soon after the approval of lentiviral gene therapy for X-ALD, Skysona was withdrawn from the European market for commercial reasons [95]. Despite years of preclinical development, withdrawal of an advanced therapy medicinal product (ATMP) from the market because of issues with reimbursements by insurance companies or governments is not a rare or isolated event. Previously, another approved HSC gene therapy for β-thalassemia (Zynteglo) was withdrawn from Germany for similar reasons. This issue is, however, not limited to ex vivo HSC gene therapy development, and other ATMPs, such as the AAV1 based vector product Glybera for the treatment of lipoprotein lipase deficiency, have also been withdrawn [96]. In addition, technical issues with the manufacturing of commercial-grade lentiviral gene product OTL-101, a lentivirus vector-transduced autologous CD34+ HSPC product with proven efficacy for the treatment of adenosine deaminase severe combined immune deficiency (ADA-SCID) [97], has caused Orchard therapeutics to completely abandon development of the product altogether.

Use of lentiviral vectors for gene corrections of HSPCs is usually performed ex vivo for a number of reasons, e.g., viral entry into the target cells is more efficient, and CD34+ HSPC populations can be selected and co-cultured in the presence of the viral vector, requiring lower viral vector batches to obtain a similar multiplicity of infection (MOI). In addition, quality control tests can be used to check CD34+ cell numbers before transplantation, perform colony assays, evaluate viral integration sites (VIS), and perform potency assays and other quality assessments. However, whereas ex vivo lentiviral therapy can be used to efficiently transduce HSPCs and provides the advantage of delivering the missing protein throughout the whole body, in vivo HSPCs transduction may circumvent the use of toxic alkylating agents. Such systems have been described using an adenovirus-based platform, based on helper-dependent adenovirus (HDAd5/35++) vectors, vectors derived from species C Ad5 serotype with Ad5 fiber replaced with affinity-enhanced Ad35 fibers [98]. More recently, HDAd6/35++ vectors derived from serotype 6 have also been exploited, because anti-Ad5/HDAd5/35++ neutralizing serum antibodies should have a lower impact on these modified vectors [99]. Therefore, some of the hurdles observed in lentiviral HSPC gene therapy, such as cytotoxicity, low uptake or cross-correction, as well as the requirements for conditioning, may be overcome with direct in vivo targeting of affected cell populations.

### 3.2. rAAV Vector Design and Serotypes for In Vivo Targeting of the Central Nervous System

In vivo delivery of rAAV can be used to directly target affected brain cell (sub)populations through a direct injection of the vector into the tissue or intravenously, since some of the AAV serotypes are able to cross the blood–brain barrier (BBB). Due to their natural AAV tropism, specific cells or cell populations can be directly targeted. Clinical studies using rAAV vectors for the treatment of leukodystrophies have shown the feasibility of this approach in recent years. In certain leukodystrophies, when the therapeutic protein is secreted to sufficiently high levels, cross-correction may occur, whereas in others where the defect lies intracellularly, the target cell requires direct correction. Limitations of rAAV-directed gene therapy depend on (1) the route of administration (RoA), which may be invasive and result in poor vector biodistribution; (2) the capsid, which determines the tropism of the vector; and (3) the dose that can be injected. Depending on the cell type of interest, promoters with cell specificity can be used to limit expression to specified cells, such as neurons, oligodendrocytes, astrocytes or microglia. Some AAV capsids, such as AAV9 and AAV.rh10, which have a predominant tropism for neurons but can also transduce astrocytes or oligodendrocytes, can be directly injected into the circulation and have the ability to cross the BBB [100,101]. Nevertheless, due to broad vector biodistribution, this method lacks efficiency, and generally less than 1% are actually transferred to the CNS. Moreover, the efficiency of intravascular delivery depends on many factors, including age, because of the maturity of the BBB, and has been shown to be more effective in young animals, such as neonatal mice. Therefore, the presence of an intact BBB is still a complicated limiting factor in the translation of systemic AAV delivery to transduce the brain in older patients. Consequently, development of novel AAV capsids through genetic engineering and selection should be used for the future generation of rAAV vectors with a higher propensity to deliver their genetic cargo to the CNS, and lower off-target transduction of non-neurological tissues.

Recent advances have been in the development of novel capsids, such as AAV.PhP.B and PhP.eB, which are more efficient at crossing the BBB, while at the same time showing reduced off-target transduction of unwanted tissues and organs, such as the liver [102,103]. However, the selection approach in mice, does not allow for the enrichment of capsids that bind well to human receptors, and thus these PhP.eB capsids bind specifically well to mouse receptor *Ly6a* [104]. Consequently, selection in non-human primates, as well as in silico prediction models using artificial intelligence (AI), may be preferred to expand the pool of available capsids that translate well into a human context [105,106]. Nevertheless, despite the presence of limited models to predict feasibility and efficacy in humans directly from preclinical animal models, an approach to develop capsids using de novo interactions with receptors that cross the BBB, such as *Ly6a* or *Ly6c*, could significantly enhance widespread vector distribution throughout the brain [107]. Another approach based on vector RNA expression, could help both the selection of AAV capsids that efficiently cross the BBB and promote cell type specificity, as has been shown for capsids with a preference for neurons and astrocytes [108]. Alternative methods that affect vector transfer efficiency may be inherent to the type of vectors used, since self-complementary vectors appear to be more efficient compared to single stranded rAAV vectors in the context of using AAV9 [109]. Commonly used delivery routes are intrathecal and intracerebroventricular (i.c.v.) injections [110,111], as well as local injections into the brain parenchyma. Risks of local injections are related to the presence of excess levels of the therapeutic protein as observed in AAV.rh10 gene therapy for mucopolysaccharidosis type IIIA, since the highest vector concentration occurs around the injection site [112], or alternatively, local injections can result in poor biodistribution affecting efficacy.

### 3.3. rAAV Vectors in Leukodystrophies

**Figure 2:**
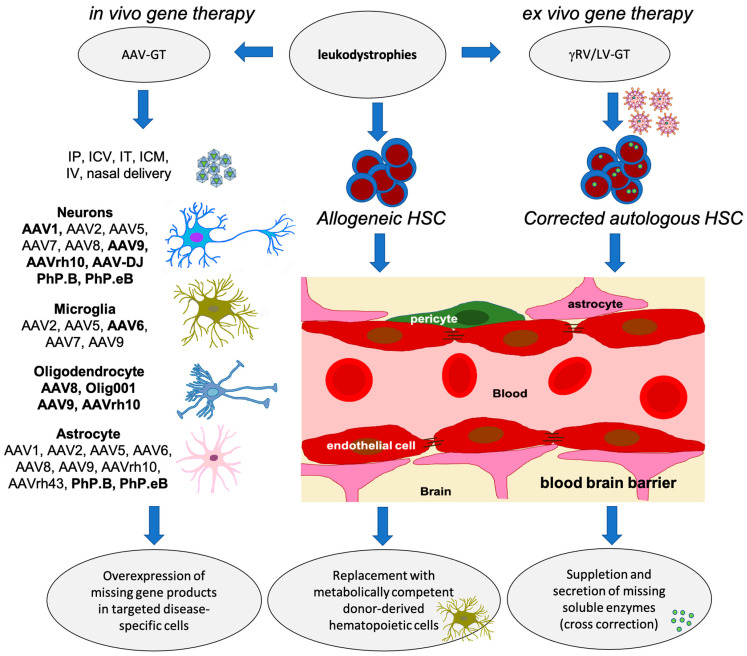
Therapeutic options for the treatment of autosomal recessively inherited leukodystrophies. The choice of AAV capsid can vary depending on the specific type of cells, brain region, age, species and intended application. Several rAAV capsid types have been identified as efficient at transducing neurons [109]. Efficient transduction of microglia, using rAAV capsids can be challenging, as microglia have unique properties and limited susceptibility to traditional AAV vectors [124]. AAV6 derivatives have shown promise in this field [125,126]. Efficient transduction of oligodendrocytes, using rAAV capsids can be challenging. Oligodendrocytes have been traditionally less susceptible to AAV transduction compared to other CNS cell types like neurons, but there are AAV capsids with known oligodendrocyte tropism [113]. While a number of AAV capsids have shown a propensity for astrocyte transduction, the efficiency may vary [127]. In vivo delivery of rAAV vectors can be obtained through multiple routes of administration (RoA), including intraparenchymal injection (IP), intracerebroventricular (ICV), lumbar intrathecal (IT), intra cisterna magna (ICM) IV injections, and even through nasal delivery. The most commonly used rAAV vector types are provided in bold. Use of gammaretroviral (γRV) or lentiviral (LV) vectors for the transfer of lacking genes is performed ex vivo after enrichment of hematopoietic stem cells. Of note: PhP.B and PhP.eB vectors are BL/6 mouse strain specific, but have been shown to function in other species as well [102,103,128,129].

To treat leukodystrophies effectively, the proper cell types need to be targeted and sufficiently reached. Different AAV capsids have been tested in preclinical models exploiting the cellular tropism needed for specific applications (Figure 2). For leukodystrophies in which the oligodendrocytes are affected, a serotype that is particularly interesting is Olig001, because of its preferential transduction of oligodendrocytes [113]. In a preclinical study for Canavan disease in adult Canavan mice, delivery of an AAV/Olig001-vector with oligodendroglial aspartoacylase (*ASPA*) cDNA through intracerebroventricular infusion into the cerebrospinal fluid (CSF) resulted in a dose-dependent rescue of ASPA activity, motor function, and a near-total reduction in vacuolation. A head-to-head efficacy comparison with astrogliotropic AAV9 highlighted a significant advantage conferred by oligotropic AAV/Olig001 that was independent of overall transduction efficiency [113]. In addition, a clinical trial using rAAV2-*ASPA* intraparenchymal delivery in 13 Canavan patients showed safety, halted neurological deterioration, and showed improved locomotor function [114]. Biodistribution may be further improved using intravenous injections with rAA9 and a constitutive active promoter. ASPA Therapeutics is currently assessing safety, tolerability and pharmacodynamic activity in pediatric Canavan patients (CANaspire; NCT04998396). To improve efficacy, the vector used in this study was also provided as dual i.v. and i.c.v. injections in one Canavan patient. After two years of treatment, the patient exhibited enhanced myelination in white matter, improved motor function, and continued to be free of epilepsy. Notably, the *N*-acetylaspartate (NAA) levels, which are increased in Canavan patients due to a deficiency of aspartoacylase, a deacetylase that catabolizes NAA, decreased at the 3-month mark and remained stable for up to four years following treatment [115]. In another phase I/II trial, Myrtelle Inc. assesses the use of the AAV/Olig001 vector designed to specifically target oligodendrocytes, through a single intracranial injection (Can-GT; NCT04833907).

Another leukodystrophy in which oligodendrocytes are predominantly affected is Pelizaeus-Merzbacher-like disease [116]. In a well-characterized *Cx32/Cx47* double knockout mouse model [117], an AAV9 vector with a myelin basic protein (MBP) promoter to transcriptionally restrict expression of gap junction protein connexin 47 gene (*Cx47*) was tested [118]. rAAV9 was i.c.v. injected at P10, and significant improvement in motor performance and coordination, as well as prolonged survival and improved tissue pathology were observed [118].

Other leukodystrophies such as vanishing white matter (VWM) disease, in which pathogenic variants in one of five subunits of the eukaryotic translation initiation factor 2 complex (*EIF2B1-5*) leads to disease, both oligodendrocytes and astrocytes are pivotal in disease pathology [119]. Targeting both these cell types would be critical for an effective gene therapy, but testing of preclinical AAV gene therapy has not been reported to date. In megalencephalic leukoencephalopathy, in which astrocytes are particularly affected, treatment of *Mlc1* knockout mice through injection of rAAV.rh10 GFAP promoter-driven *MLC1* vectors in the cerebrospinal fluid resulted in *MLC1* expression in the cerebellum and improved tissue pathology by reinstating the proper localization of the adhesion molecule GlialCAM and the chloride channel ClC-2 within Bergmann glia. Most notably, the occurrence of myelin vacuolation significantly decreased in treated *Mlc1* knockout mice, and this reduction was directly correlated with *MLC1* expression in Bergmann glia, demonstrating therapeutic effectiveness [120].

Although HSC gene therapy has been approved by EMA for the treatment of MLD patients, other gene therapy vectors, including rAAV vectors expressing *ARSA,* are also being explored. Preclinical trials using AAV vectors have been conducted to deliver the *ARSA* gene to the CNS of MLD mice and non-human primates (NHP) [121]. Similarly, clinical trials using AAV vectors for the treatment of X-ALD have focused on delivering a functional copy of the *ABCD1* gene, directly to the CNS. These studies have demonstrated successful gene delivery and increased expression of the ABCD1 protein, resulting in decreased VLCFA levels and stabilization of neurological function in some treated patients. Since GALC overexpression is toxic to HSPCs, direct delivery of the AAV vectors to the CNS to deliver the *GALC* gene and restore enzyme activity for treatment of Krabbe disease has been proposed. Efficacy and safety have been shown in both preclinical models for Krabbe disease using AAVhu68 [122], and a clinical phase I/II study that combines allogeneic HSPC with an AAV.rh10 vector delivered by a single infusion through a venous catheter inserted into a peripheral limb vein (NCT04693598, NCT05739643). However, further evaluation is necessary to assess the long-term safety and efficacy of this approach.

While these clinical studies using AAV vectors for leukodystrophies have shown promising results, it is important to note that many are still in early stages and involve a limited number of patients. Long-term follow up is necessary to evaluate the durability and safety of these treatments. Additionally, suitable in vivo models recapitulating the human diseases are still lacking for a substantial number of leukodystrophies, besides the need for optimizing the delivery methods and addressing potential immune responses to AAV vectors, which are ongoing areas of research in the field of leukodystrophy gene therapy. A schematic overview of (future) therapeutic approaches for the treatment of leukodystrophies is given in Figure 2.

Despite all the work carried out on leukodystrophy animal models, relatively few gene therapy trials have been undertaken to test efficacy in a clinical trial. The reason for this is three-fold: (1) development of gene therapy treatments depends on the availability of good animal models, as well as patient samples to test the vector delivery systems; (2) although the total number of patients affected worldwide by a type of leukodystrophy is substantial, the individual leukodystrophies are rare, and some subgroups may even be ultra-rare, such as autosomally dominant leukodystrophy (ADLD) [123], and if the patients are eligible for allogeneic HSC treatments, it may be difficult to obtain a sufficient number of patients for participation in a trial; (3) because patients are often diagnosed (too) late, they may present with advanced disease and may not show favorable responses to cell and/or gene therapy. Currently, 14 clinical trials have been registered with clinicaltrials.gov, of which five are actively recruiting patients. With the exception of two trials, all other trials are Phase I/II studies. Eight of the fourteen trials are using ex vivo lentiviral gene therapy, whereas the other trials use different types of AAV vectors. Of all the leukodystrophies, presently only gene therapy for the treatment of X-ALD, MLD, GCL and Canavan disease have reached the clinical phase. An overview of registered ongoing, recruiting and completed gene therapy trials for the treatment of primary leukodystrophies is summarized in Table 2. 

Although there are many other leukodystrophies, not all leukodystrophies are good candidates for HSC transplantation or gene therapy. Some of the reasons include absence or low physiological expression of the gene in HSPCs, absence of cross-correction even if overexpressed in HSPCs due to the nature of the protein (usually not secreted or too large for secretion), toxicity related to transgene overexpression, absence of appropriate disease models (in vitro and in vivo) to develop novel treatment strategies, and involvement of multiple genes that result in the same disease phenotype and the rarity of the disease. In addition, some leukodystrophies may sufficiently benefit from other types of treatments such as enzyme replacement therapy (ERT) or substrate reduction therapy (SRT), dietary restrictions or supporting treatments.

Microgliopathies are another rare type of leukodystrophy that could potentially benefit from HSC gene therapy. Alkylating agents can be used to create space in the CNS to facilitate engraftment and differentiation of transplanted HSPCs into microglia-like cells. However, leukodystrophies where microglial cells are the key players in disease progression are limited. Colony-stimulating factor 1 receptor (CSF1R)-related adult-onset leukoencephalopathy with axonal spheroids and pigmented glia (*ALSP*) has a primarily intrinsic microglial defect, and allogeneic HSC transplantation has been performed and shows promising results regarding the stabilization of disease progression including cognitive decline and motor function [130]. HSC gene therapy may also be suitable for Nasu-Hakola disease, in which loss-of-function mutations in the genes encoding *TREM2* or the adaptor protein through which it signals (*TYROBP*) causes extensive demyelination, astrogliosis, and activation of microglia principally in the white matter of temporal and frontal lobes and basal ganglia [131]. 

Other leukodystrophies that are of interest for HSC gene therapy encompass lysosomal storage diseases, such as Tay-Sachs disease (or Sandhoff disease) [132], in which delivery of two genes *HEXA* and *HEXB* (1:1 ratio) through rAAV administration was investigated. However, toxicity was observed in non-human primates after intracranial delivery [133]. Use of HSC transplantation has shown mitigating effects, but CNS pathology is not completely corrected, because endogenous expression remained insufficient [134,135]. Combination therapy may therefore be a necessity for adequate efficacy, as has been demonstrated on multiple occasions by combining HSC transplantation and lentiviral vector delivery [136]. For certain lysosomal storage diseases, other approaches, such as tag-technology, need to be employed. This is the case when neither endogenous expression, nor over-expression can effectively reach the CNS, but improved routing of lysosomal enzymes significantly enhances efficacy, as has been shown for mucopolysaccharidosis type II (Hunter disease) using apolipoprotein E (ApoE)-tags [137], or for Pompe disease using insulin-like growth factor 2 (IGF2) tags [138].

Some diseases that are not typically classified as leukodystrophy and develop secondary to the accumulation of unprocessed metabolic derivatives (such as deposition of lipids, cholesterol, fatty alcohols and glycogen), may display clinical signs of brain damage or leukoencephalopathy on MRI screens. Within this group, Pompe disease, caused by an absence of or mutations in the acid α-glucosidase (*GAA*) gene, and Fabry disease, caused by absence of or mutations in α-galactosidase A (*GLA*), are potential candidates for gene therapy [139]. In recent years, for both of these diseases, lentiviral [140,141,142] and rAAV [143,144,145] vector systems have been developed, some of which have now reached the clinical phase (Table 3). 

## 4. Use of Novel Technologies, CRISPR/Cas9 and Base-Editors

### 4.1. CRISPR/Cas9 Targeted Genome Editing

CRISPR/Cas9 is a widely used gene editing tool that utilizes a guide RNA (gRNA) to target specific genes and the Cas9 enzyme to make precise edits. In preclinical studies, researchers have used CRISPR/Cas9 to correct mutations associated with leukodystrophies in animal models or patient cells. Initially, homology-directed repair (HDR) was employed to change the disease mutation. This approach used to be highly inefficient, mainly because multiple components need to be delivered to one cell, e.g., the Cas9 protein, gRNA and the donor sequence. Preferably, this delivery is carried out by a single (viral) vector, but there are size limitations to most vector designs. In addition, homology-directed repair is much less efficient than non-homologous end joining (NHEJ), so most repairs result in insertions and deletions (indels). As a consequence, this can lead to null-phenotypes caused by non-sense mediated RNA decay due to premature stop codons. As an example, this approach has been used to develop a therapy for VWM. However, induction of indels resulted in a more severe phenotype [146]. Ex vivo approaches are generally more amenable to effectively incur HDR, because target cells can be enriched in small volumes, and subsequently genetically altered. This is particularly effective for genetic modification in T-cell therapy, but also for manipulating HSCs, which can be used as a source for HSC transplantation in leukodystrophy patients. Although efficiencies of donor template delivery are generally low, using AAV6 has been quite efficient in HSCs and T-cells [147,148]. Another technique that used AAV6 donor templates is called homology-independent targeted integration (HITI), which achieved ~21% stable integration of a transgene in the *IGTB2* (CD18) gene locus in HSCs [147]. Using ex vivo approaches for metachromatic leukodystrophy in patients’ cells showed that HDR-directed repair was possible [149]. An advantage of using patient’s own directly corrected cells is that transplantation complications associated with allogeneic HSC transplants can be circumvented, but correction of expression may be maximally restored to endogenous levels. In certain diseases, such as MLD, this may be insufficient for long-term efficacy. A limitation of the efficiency of HDR-directed approaches is that the majority of HSPCs are non-dividing, and stimulation of cell cycling may influence stemness of the cells, which is an unwanted trade-off for effective HDR-based approaches. In addition, induction of the DNA damage response (DDR) and p53 activation caused by double strand breaks (DSB) and exposure to recombinant AAV vector repair templates may result in reduced proliferation, engraftment, and clonogenic proliferation of edited HSPCs [150]. Side effects from this approach may be that ineffective HDR leads to more severe mutations, but also genomic translocations may cause long-term safety risks, such as contributing to genotoxicity. However, to our knowledge, there are currently no publications that have reported such incidents in preclinical models. Another option besides ex vivo applications is to directly deliver CRISPR components to target cells to edit the genes responsible for leukodystrophies; however, this has been shown to be even less efficient.

### 4.2. Base-Editing Techniques

Another CRISPR-based technology depends on the use of base editors, which is a gentler form of precise gene editing that allows for specific changes in a DNA sequence without cutting the DNA double helix. This results in a much higher editing efficiency of base editors compared to HDR (up to 200-fold), and much a lower risk for indel formation, as well as reduced formation of translocations and rearrangements [151,152]. Using base editing techniques, adenine base editors (ABEs) [153] can efficiently convert A:T into G:C, whereas cytosine base editors (CBEs) convert C:G into T:A [152]. However, the size limits delivery in vivo. Minimizing base editor complex size and developing miniature base-editors should make their use more feasible for delivery to the CNS [154]. The delivery of base editors is hampered by its large genetic cargo. Therefore, an intein-split system is often used to divide the protein sequences over two AAV vectors, which are delivered simultaneously. ALS mice treated with this approach by intrathecal delivery had a reduced rate of muscle atrophy, decreased muscle denervation, improved neuromuscular function and up to 40% fewer superoxide dismutase 1 (SOD1) immunoreactive inclusions [155]. However, it remains to been seen whether these types of constitutive active systems can be applied to human subjects. Importantly, efficient cytosine and adenine base editing efficiency was observed after administration into adult mice, effectively transducing oligodendrocytes [156]. More recently, eukaryotic-based RNA-guided endonuclease has been reported [157] to reduce the risk of immune-related responses to Cas9 protein, which has been observed in preclinical models of dystrophic dogs [158]. Despite the development of these promising techniques, correction of specific point mutations associated with leukodystrophies in both cellular and animal models needs to be further expanded for potential translation, and current base editors remain highly genome context-dependent, and are therefore not appropriate for application to a broad group of patients.

Another approach being currently investigated for leukodystrophy treatment is RNA editing, which involves the modification of RNA molecules rather than DNA [159,160]. By targeting disease-causing RNA transcripts, researchers aim to correct abnormal protein production resulting from mutations. However, for further development and use of RNA editing techniques, such as cytosine deaminases acting on RNA (ADARs) to correct RNA sequences, the potential therapeutic benefits in cellular and animal models of leukodystrophies need to be demonstrated first.

In addition to developing gene editing tools, researchers are also focusing on developing effective delivery systems to directly target the central nervous system (CNS) for the treatment of leukodystrophies. Besides viral vectors, other approaches such as nanoparticles and exosomes are being explored to deliver gene-editing components to the CNS, ensuring efficient and precise editing of target cells.

## 5. Conclusions

Gene therapies for the treatment of leukodystrophies have made the transition from non-clinical studies to clinical application. However, leukodystrophies are diverse and tailor-made therapies are required. Rather than focusing on the development of a single treatment, for most leukodystrophies, different types of vectors and cell-targeting strategies are being developed simultaneously by multiple research groups. Generally, gene augmentation has been the main advancement leading to therapeutic efficacy. In addition, molecular technologies that aim to directly correct gene mutations are being explored as well and may be applicable in certain leukodystrophies. Pitfalls, such as insufficient levels of expression, cytotoxicity, and off-target effects, as well as the need for specific cell targeting require further attention and may need some fine-tuning before these treatments reach the clinic. Furthermore, additional research is needed to assess the safety and efficacy of these gene-editing technologies, before transitioning to a clinical setting. 

Clinical trials and regulatory approvals are mandatory before these technological advances can be applied to human patients with leukodystrophies. Although only few gene therapies for the treatment of rare genetic diseases have been approved by the EMA (6) and/or FDA (2), some of these developed gene therapeutics (i.e., Skysona, Glybera, Zynteglo) have already been withdrawn after approval in Europe. This was due to reasons that do not relate to their efficacy or safety profile, but rather due to the challenge of obtaining appropriate value recognition and market access related to demanding or lacking governmental and/or insurance company reimbursements for these diseases with small, restricted and eligible patient populations. Therefore, special attention is needed for future development of viable, molecular (gene-editing/gene-addition) long-term treatment options that can reach the target population sufficiently. However, this will require intensive interaction and cooperation between the developing companies, patient representatives, insurance companies and governments. 

## Figures and Tables

**Table 3:** Overview of gene therapy clinical trials for secondary leukodystrophy.

Clinical Trial	Title/Year	Country	Vector/ Transgene	NTC No
Ex vivo gene therapy				
Phase I	Gene Therapy for Gaucher’s and Fabry Disease Using Viruses and Blood-Forming Cells (1988–2022)	USA	RV-aGLA	NCT00001234
Phase I active	Autologous Stem Cell Transplantation of Cells Engineered to Express Alpha-Galactosidase A in Patients With Fabry Disease (2016–2024)	Canada	LV-aGLA	NCT02800070
Phase I/II terminated	Open Label, Study Of Efficacy and Safety Of AVR-RD-01 for Treatment-Naive Subjects With Classic Fabry Disease (2018–2022)	USA	LV-hGLA (AVR-RD-01)	NCT03454893
Follow-up terminated	Long-Term Follow-up Study of Subjects With Fabry Disease Who Received Lentiviral Gene Therapy in Study AVRO-RD-01-201 (2019–2023)	Australia	LV-hGLA (AVRO-RD-01-201)	NCT04999059
In vivo gene therapy				
Phase I/II completed	Safety Study of Recombinant Adeno-Associated Virus Acid Alpha-Glucosidase to Treat Pompe Disease (2010–2015)	USA	rAAV1-CMV-hGAA	NCT00976352
Phase I/II completed	Re-administration of Intramuscular AAV9 in Patients With Late-Onset Pompe Disease (AAV9-GAA_IM) (2017–2021)	USA	rAAV9-DES-hGAA	NCT02240407
Phase I/II	AAV2/8-LSPhGAA (ACTUS-101) in Late-Onset Pompe Disease (2018–2026)	USA	AAV2/8-LSP-hGAA (ACTUS-101)	NCT03533673
Phase I/II terminated	A Fabry Disease Gene Therapy Study (MARVEL1) (2019–2023)	USA	AAV (FLT190)	NCT04040049
Phase I/II recruiting	Dose-Ranging Study of ST-920, an AAV2/6 Human Alpha Galactosidase A Gene Therapy in Subjects With Fabry Disease (STAAR) (2019–2024)	USA	AAV2/6—hGLA (ST-920)	NCT04046224
Phase I/II	A Gene Transfer Study for Late-Onset Pompe Disease (RESOLUTE) (2020–2027)	USA	AAV-rh74-GAA (SPK-3006)	NCT04093349
Phase I/II recruiting	Gene Transfer Study in Patients With Late Onset Pompe Disease (FORTIS) (2020–2029)	USA	AAV8-GAA (AT845)	NCT04174105
Follow-up active	A Long Term Follow-Up Study of Fabry Disease Subjects Treated With FLT190 (2020–2030)	Germany, UK	AAV (FLT190)	NCT04455230
Phase I/II recruiting	An Open-label, Phase 1/2 Trial of Gene Therapy 4D-310 in Adults With Fabry Disease (2020–2027)	USA	AAV (4D-310)	NCT04519749
Follow-up active	Long-Term Follow-up of Subjects Who Were Treated With ST-920 (2021–2029)	USA	AAV2/6—hGLA (ST-920)	NCT05039866
Phase I/II recruiting	Clinical Exploration of Adeno-associated Virus (AAV) Expressing Human Acid Alpha-Glucosidase (GAA) Gene Therapy for Patients With Infantile-onset Pompe Disease (2022–2025)	China	AAV9-hGAA (GC301)	NCT05567627
Phase I/II active	4D-310 in Adults With Fabry Disease and Cardiac Involvement (2022–2028)	Australia	AAV(4D-310)	NCT05629559
Phase I/II recruiting	Evaluation of the Safety and Efficacy of Infantile-onset Pompe Disease Gene Therapy Drug (2023–2024)	China	AAV9-hGAA (GC301)	NCT05793307

## Data Availability

Not applicable.
